# Indoor Air Quality Monitoring and Characterization of Airborne Workstations Pollutants within Detergent Production Plant

**DOI:** 10.3390/toxics10080419

**Published:** 2022-07-26

**Authors:** John Stephen Gushit, Salamatu Uba Mohammed, Haruna Musa Moda

**Affiliations:** 1 Department of Chemistry, University of Jos, Jos 930001, Nigeria; gushitj@unijos.edu.ng (J.S.G.); salamatu.mohammed@nascogroup.net (S.U.M.); 2 NASCO Household Products Limited, 44 Yakubu Gowon Way, Jos 930001, Nigeria; 3 Department of Health Professions, Manchester Metropolitan University, Manchester M15 6BG, UK

**Keywords:** monitoring, characterization, indoor air quality, detergent plant

## Abstract

The indoor air quality (IAQ) of five workstations within a detergent production unit was monitored. Particulate matter (PM) was measured using a gravitational settlement method, and later characterized. To ascertain the quality of indoor air within the workstations, which could directly or indirectly affect the health and performance of the workers, a physical inspection of the plant premises was undertaken. The mean value of the following air-quality parameters; particulate matter(PM_2.5_), particulate matter (PM_10_), formaldehyde (HCHO), volatile organic compounds (VOCs), carbon dioxide (CO_2_), temperature (T) and percent relative humidity (%RH) were obtained within the range of 24.5–48.5 µg/m^3^, 26.75–61.75 µg/m^3^, 0.0–0.012 mg/m^3^, 0.09–1.35 mg/m^3^, 1137–1265 ppm, 25.65–28.15 °C and 20.13–23.8%, respectively. Of the particulate matter components characterized, sodium oxide (Na_2_O)—25.30 mg/m^3^, aluminum oxide (Al_2_O_3_)—22.93 mg/m^3^, silicon dioxide (SiO_2_)—34.17 mg/m^3^, sulfur trioxide (SO_3_)—41.57 mg/m^3^, calcium oxide (CaO)—10.94 mg/m^3^ and iron III oxide (Fe_2_O_3_)—19.23 mg/m^3^, were of significance. These results, compared with international standards for industrial indoor air quality, suggest that indoor air contamination emanating from the chemicals used in production workstations is traced to the design of the plant structures and the activities carried out within the workstations.

## 1. Introduction

Indoor air quality (IAQ) is a measure of how clean the air is inside the buildings in which we work, live, or play. This is influenced by factors that include building material types and furniture, the building’s ventilation system, the chemicals that are used and created by the activities carried out within the building, etc. [1]. According to the United States Environmental Protection Agency (USEPA) [2], the indoor air we breathe can put us at risk for health-related problems, since most of our time is spent indoors. Previous studies have reported that industrial exposure to surfactants and detergents is associated with asthma and other adverse health effects [3,4,5]. Health risks such as irritation to the eye, nose, and throat, coughing, asthma and cancer, are based on short- and long-term exposures to pollutants in the indoor air, as well as the physiological health status of the population at risk, and the thermal conditions within the environment. In extreme cases, exposure to indoor air pollutants (IAPs) can even cause death [6,7]. Aside from inhalation, these IAPs can enter the body through hand-to-mouth contact and dermal exposure [7].

Past studies have reviewed the harmful effects of exposure to some IAPs; particulate matter (PM) pollutants such as suspended dust and reactive products from industrial chemicals were found to have association with work-related respiratory incidences such as pneumonia and bronchitis [8]. Heavy metals associated with PM can be cumulative in nature, causing hepatic cell destruction and lung inflammation [9]. Biological pollutants such as molds, viruses and bacteria which grow in damp and warm environments have caused sneezing, coughs, and shortness of breath, and triggered allergic reactions such as rhinitis [10]. Building materials, furniture, cleaning products and personal care products are some indoor sources of VOCs such as benzene, limonene and trihalomethanes. Some of these VOCs are known or suspected to cause cancer in humans [7,11]. Carbonyl compounds, as described by Diodu et al. [12], affect human health as primary irritants of the mucous membranes of the eyes, the upper respiratory tract, and the skin. This also occurs indoors as secondary emissions from pesticides, consumer products and building materials [7]. Similarly, carbon monoxide (CO) is found indoors in fumes because of incomplete combustion from burning fuels in small engines, gas ranges and furnaces. Common symptoms of CO poisoning are headache, dizziness, weakness, and chest pain. It is an odorless, colorless gas that can kill [8]. On the other hand, carbon dioxide (CO_2_) gas is not considered to pose serious health challenges to occupants at elevated concentrations. However, it can cause drowsiness, lethargy and gives a general sense that the air is stale. The indoor sources of CO_2_ are associated with human respiration and combustion activities [7,13]. Other IAPs include ozone, explosive gases such as methane, nitrogenous compounds, radioactive particles, and other gases [14,15].

The present study was undertaken after numerous complaints from employees of the detergent plant received by the health, safety, and environment (HSE) committee, due to inhalation of suspected substances present in the indoor air that led to workers experiencing irritation of the nose, throat, and eyes, leading to sneezing, coughs and difficulty breathing. This was especially prevalent after a prolonged stay in the workstations where raw materials associated with detergent production were handled, with resultant releases of gases, vapor, and particulate matter (PM) into the immediate and extended environments, which acts as primary source of IAPs. Research works on indoor air quality concerning detergent industries are very sparse in Nigeria, if available at all; as such, the study monitored and characterized the indoor air of workstations within a detergent plant to ascertain the air quality.

## 2. Materials and Methods

### Study Design and Measurements

**Figure 1:**
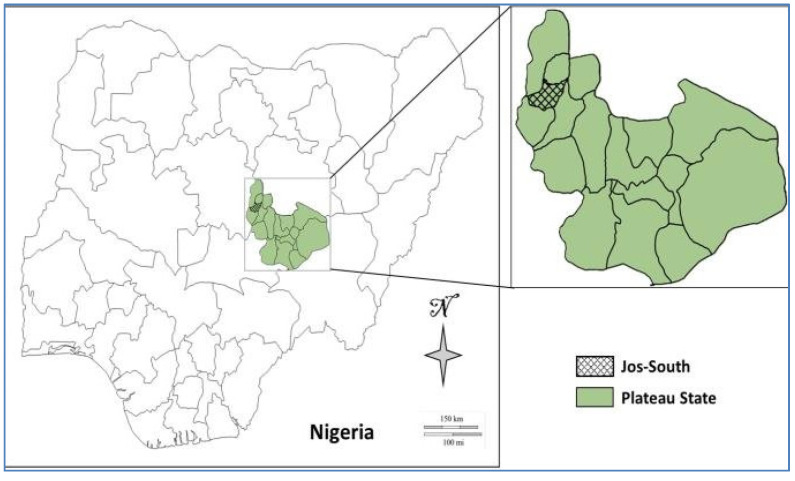
Map of Nigeria Showing the Study Area within Plateau State.

**Figure 2:**
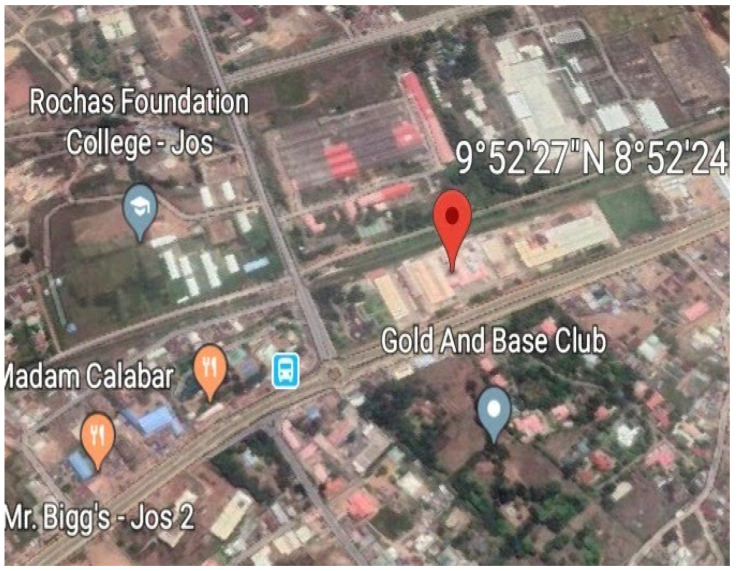
Google Earth (2019) Satellite Map Showing NHPL Jos Plateau State Nigeria.

The study area is the detergent unit of NASCO Household Products Limited (NHPL) Jos, located in the Jos South Local Government Area of Plateau State, Nigeria (Figure 1) [16]. It is geographically enclosed within latitude 9°52′27″ N and longitude 8°52′24″ E [17]. NHPL is one of the several distinct business divisions of the NASCO Group of Companies. It was established in 1973 as the detergent, soap, and cosmetic unit of the Group, manufacturing a variety of products as well as industrial chemicals, providing employment and livelihood to hundreds of employees across Nigeria [18,19]. The plant is located 80 m from a busy road, with heavy carriage of assorted vehicles and 2 km from a major stream, which is the water source for irrigation and other agricultural purposes (Figure 2).

**Figure 3:**
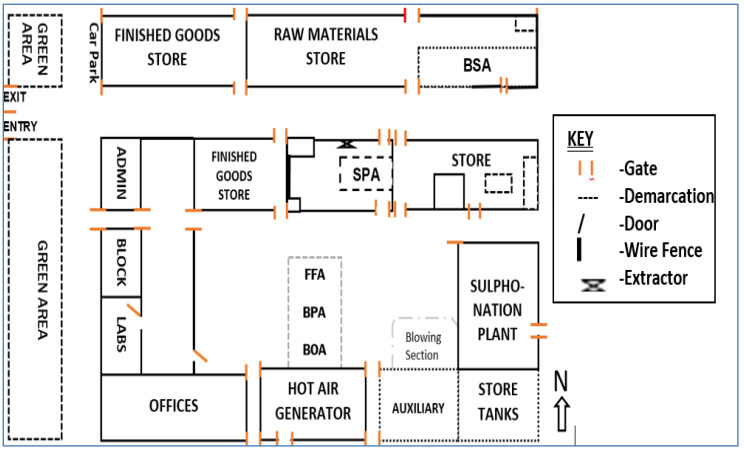
Sketched Layouts of the Workstations.

The workstations were labeled the bulk pack area (BPA), bulk oversize area (BOA), first floor area (FFA), small pack area (SPA) and batch slurry process (BSP), according to the activities carried out within them. These workstations, which house a minimum of 59 and maximum of 137 workers (Table 1 and Table 2), are enclosures, each within a larger hall of not less than 800 m^2^ in area. The hall housing the BPA, BOA and FFA shares a wall lengthwise to the hall housing the SPA, while the hall housing the SPA is parallel to that housing the BSP. The FFA occupies an area of 200 m^2^ on the first floor of the building, directly above the BPA and BOA as stated in Table 1. These workstations are naturally ventilated via vent openings on the lengthwise sides, doors, and large gate, as shown in Figure 3. The plant has assorted brands of products such as Brytex, Bonus and Action (detergents) formulated from chemical substances such as linear alkyl benzene sulfonate, sodium salts, zeolites, speckles, sodium carboxy methyl cellulose, perfumes of interest (mostly citrus based), copolymers, enzymes, and optical brighteners. The criteria used to assign the workstations hinged upon the functionality of the workstations at the time of the research and the involvement of two or more persons at each workstation during normal operations. Selection and measurements were carried out following the Occupational Safety Health Administration (OSHA) guidelines on indoor air quality investigation [20] and that of the National Environmental Standards Regulations Enforcement Agency (NESREA) (schedule XI regulation 18(3)) of Nigeria [21]. Measurements were carried out between 8 of October and 9 November 2018, which was within the peak period of production in the industry. An eight (8)-hour sampling period was adopted (schedule VI regulations 14, 19) between the hours of 9:00 a.m. and 5:00 p.m. which are the peak office hours. The natural ventilation makeup of the plant was considered sufficient and adequate for supplying fresh air to dilute pollutants for acceptable IAQ [20]. For this reason, air flow rates/infiltration rates through the workstations were not measured in this study.

Spot measurements for IAQ parameters were taken using a calibrated real-time air-monitoring device (Air Master AM7) [22]. To simulate the average human breathing zone, the monitoring device was positioned at a height of 1.7 m close to each monitored workstation where activities are undertaken (Figure 3). Surrogate measurements were carried out at a four time slots [21] and obtained values were recorded.

A modified method of gravitational settlement was used to collect PM for characterization, bearing in mind that PM concentration is affected by meteorological, physical, and chemical factors [23]. The test and re-test of reliability measurements were carried out prior to sampling to ascertain the reliability and validity of the method [24]. In this method, a high-grade Whatman 542 hardened ashless filter paper of 124 mm diameter was put into a 200 mL volume, 155 mm diameter porcelain crucible after preconditioning for 24 h. These were suspended on a platform about 1.2 m from the floor, corresponding to the breathing zone level of the workforce [21]. The sampling frequency was thrice a week for each sampling site. At the end of each 8 h, the filter paper containing settled PMs was collected and kept in labeled envelopes, sealed, and stored in a plastic carrier. PMs collected for each site were stored separately in sterile bags [23] and maintained at room temperature prior to the characterization using Energy Dispersive X-ray Fluorescence (ED-XRF Model X-Supreme 8000), a multi-sample bench analyzer. The equipment was calibrated using standard references for each element supplied by the National Institute of Standards and Technology (NIST) to obtain the X-ray intensity for each element of interest. It functions on the principle of excitation at an atomic level. As the elements return to their initial state, they emit characteristic X-ray photons, which can be detected and quantified. A calibration line is achieved and used to perform quantitative analysis [25].

A mineral analysis method covering a data series of the element’s sodium to Chlorine, within the X-ray energy range of 22 KV was used. About 15 g of each sample was taken into a 40 mm diameter plastic sample cup and covered with plastic support films to ensure a flat homogeneous surface. These “compact” sample cups were placed into the sample carousel of the instrument and allowed to run for 5 min. Calculated results were displayed on the monitor and the precision was calculated from repeated measurements of the NIST standards [25,26,27].

## 3. Statistical Analysis

The Statistical Package for Social Scientist (SPSS, Version 21) was used in the analysis of the data obtained, which provided both descriptive and inferential meaning to the study. The decision *p* < 0.05 was considered significant at 95% confidence level. Pearson moment correlation analysis was also performed within and between the variables at a significant level of 0.01.

## 4. Results

### 4.1. Visual Assessment of Workstation

Based on the physical observation undertaken, several staff were observed using recommended PPE inappropriately at some point, thereby increasing the exposure likelihood of fugitive pollutant arising from apparatus during use, either due to faulty equipment, leakage, or other unforeseen mishaps. In addition, existing ventilation equipment were in operation, although it was observed that a mobile LEV is needed to cater for certain activities, such as measurement and emptying of bulk materials to minimize dispersal of escaped materials into the immediate work environment.

### 4.2. PM_2.5_, PM_10_, Formaldehyde and Temperature—The FFA

From Table 3, the FFA presented the highest concentrations for PM_2.5_, PM_10_, formaldehyde (HCHO) and temperature (T) as 48.5 ± 6.25 µg/m^3^, 61.75 ± 9.53 µg/m^3^, 0.0125 ± 0.00 mg/m^3^ and 28.15 ± 0.1 °C, respectively. The characteristic of the sampling point area is that it is closest to corrugated zinc roofing, which is in direct contact with the sun’s rays. This platform is separated from the auxiliary section beside it by large vents, in which alkaline sodium silicate is being dissolved at a high temperature (1200 °C) in an autoclave and stored in high-volume tanks for use. The platform also holds varieties of perfumed powders. These activities create steam, heat, mist, and dust [22,24], which informs the highest PM concentrations and temperature recorded. PMs have been found to increase the prevalence and incidences of bronchitis, cough, and deficiency in lung function; PM_2.5_ can penetrate deeply into the alveolar region of the lungs [28]. The International Agency for Research on cancer has classified PMs as carcinogenic to humans [29]. Concentrations of PM_2.5_ for the workstations were thus: FFA (48.50 µg/m^3^) > BPA (44.00 µg/m^3^) > BSP (38.25 µg/m^3^) > SPA (28.75 µg/m^3^) > BOA (24.50 µg/m^3^). Meanwhile, the concentrations for PM_10_ followed a similar trend for PM_2_._5,_ indicating the concentrations of 61.75 µg/m^3^ > 54.00 µg/m^3^ > 45.25 µg/m^3^ > 33.75 µg/m^3^ > 26.75 µg/m^3^ for FFA, BPA, BSP, SPA and BOA, respectively (Table 3). In addition to the BPA, which had the next large concentrations of PMs and formaldehyde after the FFA, is the BOA located to the south of the area, which is separated only by pillars. Large-particle-sized (unscented) detergent with high moisture content directly from the drying tower, with a dropping temperature as high as 60 °C, is collected into bulk packaging for recycling. To the south, the BOA is separated by a vented wall from the hot air generator (HAG) house. The hot, moist, unscented large detergent particles may have resulted in the lowest levels of PM_2.5_ and PM_10_ for 24.50 µg/m^3^ and 26.75 µg/m^3^, respectively, recorded for the BOA workstation. The high temperature of the BOA, similar to that of FFA, contributes to the combustion of products of hydrocarbons in the detergent and the fuel from the HAG. Based on an 8 h time-weighted average (TWA), the PM concentrations of all the work stations lie within permissible exposure limits (PELs) of the Occupational Safety and Health Administration (OSHA) [29] reported concentration of PM_2.5_ for 50 µg/m^3^ and PM_10_ for 150 µg/m^3^ and that of NESREA [21] with 180 µg/m^3^ for PM_10_ only, as presented in Table 4. Statistical analysis of PM_2.5_ and PM_10_ showed a strong correlation, which indicated that a rise in the value of one led to a corresponding rise in the value of the other.

Formaldehyde (HCHO) is a combustion byproduct of unvented furnaces and water heaters [30] such as the autoclave and the drying tower. Research has shown that the emission of HCHO from materials is positively related to temperature and humidity, which must have informed its out-gassing from synthetic perfumes that likely contain terpenes, limonene, and the like [28,30] constituted in the powders. However, the humidity of the FFA was not the highest of the workstations. Exposure to HCHO vapors can produce short-term symptoms such as headaches, itchy and/or burning eyes and nose, asthma, respiratory difficulties, depression, insomnia, and mental confusion. People with asthma and hyper-reactive airways are more susceptible to formaldehyde [28]. HCHO was not detected in the SPA, which demonstrated that it had no significant effect on the IAQ of the investigated workstations. In all the workstations, PELs for formaldehyde of OSHA (0.75 mg/m^3^) [31] and NESREA (0.10 mg/m^3^) [21] were not exceeded. The values were well below 50% of the PELs (Table 4).

The body temperature rises with increasing radiance temperature from surfaces involved in the release of heat within an environment [32]. This is the case when following the characteristics of the FFA, as stated above. In addition to this, human body exchanges heat with the environment through convection, radiation, evaporation, and respiration, which could be a contributory factor to the higher temperatures recorded for all the workstations with FFA (28.15 °C) > BOA (27.60 °C) > BSP (27.55 °C) >SPA (26.95 °C) > BPA (25.65 °C). Higher temperatures than what is considered moderate (22–25.50 °C) have been found to have profound effects on thermal comfort in an indoor environment, leading to heat exhaustion, changes in heartbeat rates, accuracy in brain functions and response time to stimuli. Advanced levels of temperature difference could lead to death [21,32,33]. Temperature levels of all the workstations exceeded the PEL of OSHA (25.50 °C) [34] and NESREA (24.44 °C) [21]. Providing thermal balance, though not necessary (as it differs from person to person due to age, sex, body mass index (BMI), etc.), may help improve the performance of indoor employees [32,33].

### 4.3. Volatile Organic Compounds (VOCs)

**Figure 4:**
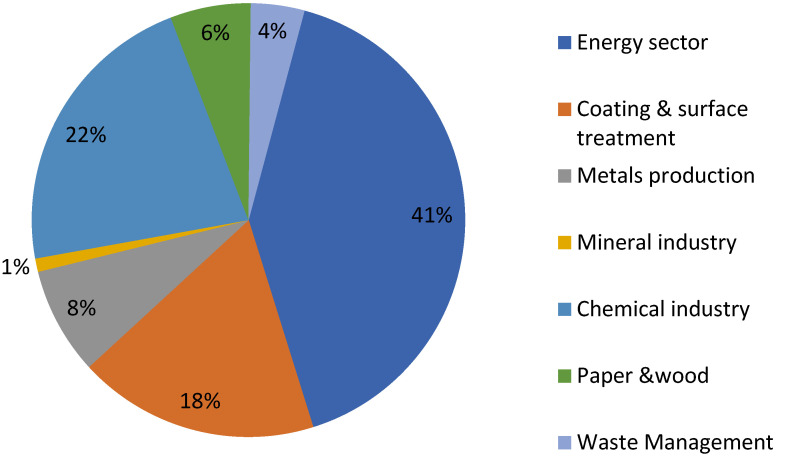
Sectors involved in High VOCs Emissions for the EU-27.

In this research, specific VOCs were not targeted. The values of VOCs obtained followed the trend BPA > BOA > FFA > SPA > BSP, corresponding to concentrations of 1.35, 0.98, 0.93, 0.27 and 0.09 mg/m^3^, respectively. The BPA with the highest value of VOCs holds a minimum of nine persons at any time of shift. VOCs concentration of 1.35 mg/m^3^ is high when compared with the Australian National Health and Medical Research Council maximum recommended indoor limit for VOCs of 0.500 mg/m^3^ [35]. The BPA is where the products are packed into the bags almost directly from the drying tower, during which the volatile compounds are emitted into the workstation. The effect is reflected in the high number of complaints associated with VOCs exposure (Table 2). This goes on to provide indication of future health impacts to workers health and wellbeing aligning with a report that exposure to low levels of VOCs is associated with higher levels of asthma, cancer, and other adverse health effects [36,37,38]. As described earlier, to the south of the area of the BPA is the BOA, and above these two workstations is the FFA. The detection levels for BPA, BOA and FFA were in abundance, with over 37% of the PELs of both OSHA [28] and NESREA [21] of 0.75 and 0.60 mg/m^3^, respectively. However, the values from this research were found to be lower than those recorded in an earlier finding by Mukurarinda [11], who reported that indoor VOCs concentrations are generally found in higher levels than the ambient outdoor levels and are majorly emitted from building materials, combustion processes, consumer products and personal care products. According to the European Environment Agency, the main sectors involved in high VOCs emissions for the EU-27 are solvent and product use with 41%, followed by the chemical industries (such as NHPL) with 22% as seen in Figure 4. As a household cleaning agent, detergent powder releases VOCs into the indoor environment even in storage, majorly from the constituted perfumes. The extent of the health effects of VOCs depends on factors such as the level of exposure and length of time of exposure; however, immediate symptoms soon after exposure include eye and respiratory tract irritation, headaches, dizziness, visual disorder, and memory impairment [39]. Source removal, source control and natural ventilation in buildings are some of the appropriate approaches that can reduce the risk of hazardous exposure to air pollutants found indoors [28].

### 4.4. Carbon Dioxide (CO_2_) and Relative Humidity (RH)

**Figure 5:**
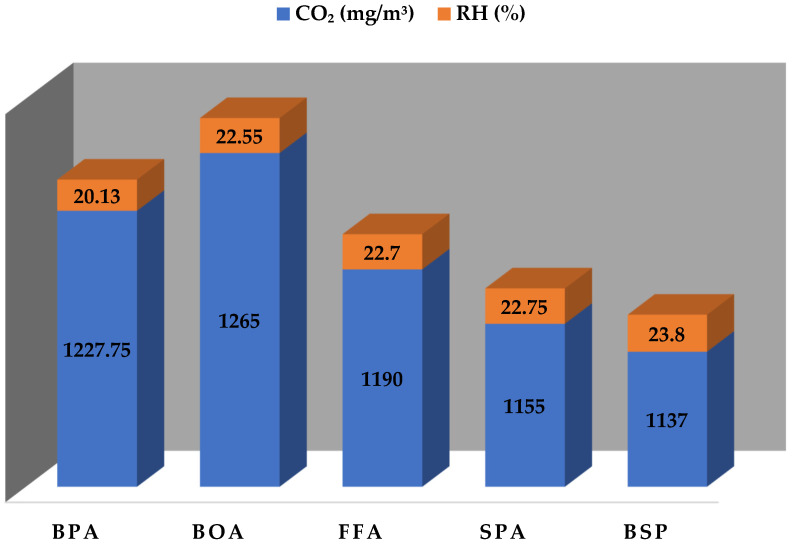
Monitored CO_2_ concentration and RH across the workstations.

The highest concentration of CO_2_ was recorded for the BOA of 1265 ± 55.82 mg/m^3^ with RH of 22.55 ± 0.20%, while the lowest CO_2_ concentration was recorded for the BSP of 1137 ± 2.00 mg/m^3^, with the highest value RH of 23.8 ± 0.52%. From the former, it could be deduced that there is a small degree of correlation between the CO_2_ concentration and the %RH of the workstations, as seen in Figure 5. As the RH concentration increased, the CO_2_ concentration decreased for the stations.

From this behavior, it can be inferred as the RH of the workstations increased towards the acceptable limits between 60% and 70%, it is likely to impact the concentration of CO_2_ from low concentrations to concentrations in which the perceived air quality acceptability does not significantly change, as described [40]. This behavior was attributed to the natural ventilation available to each of the stations, as described earlier (Table 1 and Figure 3). The concentrations of CO_2_, being a byproduct of complete combustion and human metabolism, are often used to indicate if adequate air is being supplied to a building or not. Moderate to high levels (350–1000 ppm) can cause headaches and fatigue, and higher concentrations (1000–2000 ppm) can produce nausea, dizziness, and vomiting. Difficulty breathing, sweating, tiredness, increased heart rate and loss of consciousness can occur at extremely high concentrations (2000–5000 ppm or more) [41]. In this study, CO_2_ concentrations were found to be well below the PEL of the regulatory bodies of 5000 mg/m^3^ by OSHA [42] and 10,000 mg/m^3^ by NESREA [21]. The RH for all sampled workstations was around 39% lower than the lowest PEL; that is, 60.00% for OSHA [32] and 70.00% for NESREA [21]. It was concluded the low RH was also attributed to the season in which the sampling was performed (October–November), which is usually cool and dry for Jos and its environs [43]. Though the relationship between health, indoor air humidity and pollution is complex and challenging, several studies in the office environment have shown associations between low RH (5–30%), with increasingly prevalent complaints about perceived dry and stuffy air, and sensory irritation of the eyes and upper airways [44,45,46]. In a study of 484 office workers and 21 greenhouse employees in normal and well-ventilated office buildings, though dependent on psychosocial and environmental factors, low RH produced a few percentage reductions in visual acquisition for certain office tasks among young students that were exposed for 4 h, an effect that was expected to be more pronounced among the elderly. This could be a likely scenario in the case of this study.

### 4.5. Particulate Matter Characterization

**Figure 6:**
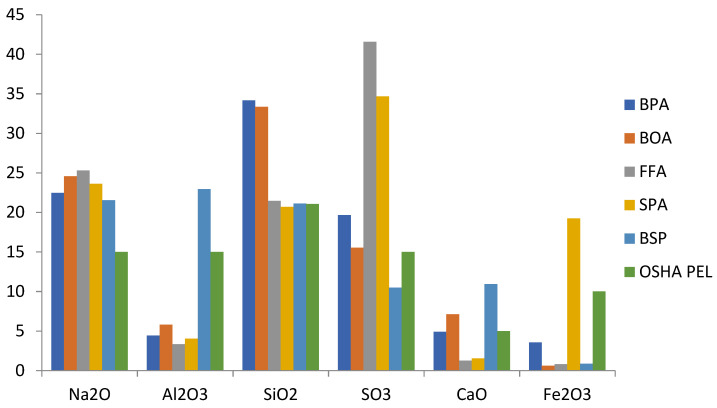
Concentrations (mg/m^3^) of Characterized Particulate Matter vs. OSHA PELs.

Particulate matter characterization, as shown in Table 5, revealed fifteen (15) components comprising of Na_2_O, MgO, Al_2_O_3_, SiO_2_, P_2_O_5_, SO_3_, Cl^−2^, K_2_O, CaO, TiO_2_, Cr_2_O_3_, Mn_2_O_3_, Fe_2_O_3_, ZnO and SrO. These values are weight percent concentrations based on three days (each day for 8 h). The concentrations in milligrams per meter cube (mg/m^3^) obtained for an 8 h TWA for each particulate matter [47,48,49] are provided in Table 5, and the values obtained are compared with OSHA PEL [50] as total dust, metal, or fumes. There are particulates which are neither regulated nor established, for which OSHA has provided a single 8 h TWA PEL of 15 mg/m^3^ measured as total particulate [50]. From Table 6, MgO, P_2_O_5_, Cl^−^, K_2_O, TiO_2_, Cr_2_O_3_, Mn_2_O_3_, ZnO and SrO are of insignificant concentrations when compared to their OSHA PEL. The remaining Na_2_O, Al_2_O_3_, SiO_2_, SO_3_, CaO and Fe_2_O_3_ (Figure 6) are of significance and are discussed.

### 4.6. Sodium Oxide (Na_2_O)

The FFA contained the highest value for Na_2_O, with 25.30 mg/m^3^ > BOA (24.59 mg/m^3^) > SPA (23.62 mg/m^3^) > BPA (22.46 mg/m^3^) > BSP (21.54 mg/m^3^), in that order. It is observed that the concentrations of Na_2_O in all the workstations exceeded the OSHA PEL [50] of 15 mg/m^3^. Na_2_O mostly originates from the dissolved alkaline silicate and other aluminosilicate materials that are added to improve detergent performance. The FFA holds warm (>50 °C) detergent slurry crutchers stirring at high speed. It also holds all the varieties of powder, and its proximity to the silicate-dissolving section makes it prone to having the highest concentration of Na_2_O. Na_2_O causes a sore throat, cough, a burning sensation, and shortness of breath on inhalation. It also causes redness, pain and burns to the eyes and skin [51]. It is contacted via all routes of exposure and reacts violently with water to produce sodium hydroxide (NaOH) [51,52]. When in contact with the moist environment of the respiratory and digestive systems, the NaOH formed—in sufficient amounts—can hydrolyze proteins in the tissue, causing burns and leading to tissue injuries. This can cause accumulation of fluid in the lungs, which could lead to perforation of the gastrointestinal tract. Upper airway obstruction may occur on exposure to it [52].

### 4.7. Aluminum Oxide (Al_2_O_3_)

Preparation of all detergent brands begins at the BSP. There is delivery of needed raw materials such as the aluminosilicate materials in larger quantities for bulk pack powders. This was the scenario as at the time of sampling; hence, the highest concentration of Al_2_O_3_ was recorded at the BSP of 22.93 mg/m^3^ which is above the OSHA PEL of 15 mg/m^3^ [50]. All the other workstations recorded less than 15 mg/m^3^. Aluminum oxide (Al_2_O_3_) dust causes irritation to the eyes and skin on contact. It is absorbed into the body by inhalation of its aerosol, causing nose, throat, and lung irritation. In addition, it also causes coughing, wheezing and shortness of breath. Long-term or repeated exposure causes lung damage and may have an effect on the central nervous system [53,54].

### 4.8. Silicon Dioxide (SiO_2_)

The results for SiO_2_ measured for each workstation revealed different concentrations, where BPA (34.17 mg/m^3^) > BOA (33.35 mg/m^3^) > FFA (21.45 mg/m^3^) > BSP(21.12 mg/m^3^) > SPA (20.70 mg/m^3^), respectively (Table 6). Based on physical examination of activity types taking place at each workstation, BPA, and BOA higher concentrations of silica correlate with the formulation (bulk pack production) process taking place at these sites, as it is often supported by a higher dose of silicate compared to those for small packs. The FFA and BSP experienced a slight rise in concentrations above the OSHA PEL [50] of 21.06 mg/m^3^, while that of the SPA is within the limit. OSHA [55] classified silica as a human lung carcinogen. Breathing crystalline silica dust causes formation of scar tissue upon entering the lungs, which affects lung functions and makes a person more susceptible to other lung infections such as tuberculosis. The lung disease silicosis is caused by breathing in silica dust, and its symptoms include shortness of breath, fatigue, and chest pain. Long-term exposures lead to weight loss. In acute form, it leads to respiratory failure, which often results in death [55].

### 4.9. Sulfur Trioxide (SO_3_)

Sulfur trioxide is less likely to be found in air, but for a short period. It is formed slowly in the air from sulfur dioxide (SO_2_) and rapidly takes up water (moisture) to form sulfuric acid (H_2_SO_4_) once inhaled. The latter causes nose irritation, and on entering the respiratory tract, it affects the tract, tissues, eyes, and the gastrointestinal tract [56]. Concentrations of SO_3_ in the workstations are: FFA (41.57 mg/m^3^) > SPA (34.68 mg/m^3^) > BPA (19.67 mg/m^3^) > BOA (15.52 mg/m^3^) > BSP (10.48 mg/m^3^) (Table 6). Inspection of the plant layout revealed, a sulfonation plant was stationed within the hall housing the BPA, BOA, FFA and SPA (Figure 3). The SPA is only enclosed by a wall (not complete to the roof) within the main hall housing of the workstation. The sulfonation plant uses sulfur, which is melted in oxygen to produce SO_3_ before further reactions to form the sulfonic acid. The results recorded for the BSP (10.48 mg/m^3^) which is about 45 m in parallel away from the sulfonation process, suggests leakages around the sulfur melter, converter and the reacting vessels, leading to the escape of SO_2_/SO_3_ gases. Apart from the BSP, all the other workstations within the hall housing the sulfonation plant have higher concentration values than the PEL of 15 mg/m^3^ by OSHA [50].

### 4.10. Calcium Oxide (CaO)

As in the case of Al_2_O_3_, high values of CaO were recorded at the BSP (10.94 mg/m^3^), followed by the BOA (7.10 mg/m^3^) then, the BPA (4.90 mg/m^3^) as a result of the aluminosilicates added to bulk production for improved performance. From the OSHA [50] PEL, the BPA concentration is at the brink of falling over PEL, while the BSP and BOA concentrations have exceeded the PEL of 5 mg/m^3^. Calcium oxide is contacted via all routes of exposure, causing a burning sensation, cough and shortness of breath, sore throat, skin redness and blurred vision. It causes abdominal cramps, vomiting and diarrhea. Prolonged or repeated contact and inhalation may cause skin dermatitis, nasal ulceration, and perforation of the nasal septum [57].

### 4.11. Iron (iii) Oxide (Fe_2_O_3_)

Result for Fe_2_O_3_ measured within the SPA plant area was 19.23 mg/m^3^. This value when compared with OSHA standards, was found to have exceeded the PEL for Fe_2_O_3_ fumes (10 mg/m^3^) [48]. This result is not surprising, as it is an area with large detergent holding tanks, some of which are made of the iron metal. In addition, small pack detergents are packed at the SPA, and the tanks are agitated vigorously to deliver their detergent powders from time to time. No categorical statements have been made on the carcinogenicity of iron oxides in humans. OSHA agrees that any occupational exposure that causes foreign substance to lodge in the body tissues is undesirable. Iron oxides are poorly soluble; however, it cannot be excluded that small amounts of soluble iron (ii) or Iron (iii) are also present in the lungs after inhalation. These can trigger genotoxic and carcinogenic changes due to simultaneous exposure to other toxic compounds [58,59].

Findings from the research recommend that extractors be installed at various workstations to eliminate these pollutants to help keep the workforce safe. In addition, the workstations should be remodeled to allow for adequate cross ventilation. Based on the existing building design, there is a need for installation of further ventilation technology to accommodate the need within each work environment without necessarily tampering with the architecture of the plants. Considering the lack of national standards in Nigeria for IAQs, there is a need to adopt international reference IAQ standards that should guide the of monitoring air quality within the work environment to minimize work-related illness and enhance productivity with the organization. To the best of our knowledge, this is the first study that considers the indoor air quality within detergent manufacturing plants in Nigeria, as such data contained herein have helped to highlight the likely impact of indoor air quality on employees’ health and wellbeing in similar work environments throughout the country. In addition, the work further draws attention of regulatory bodies to oversights regarding their responsibilities around safety and wellbeing. It will ensure that adequate monitoring of indoor air quality is maintained across industries in the country, while ensuring health surveillance records are maintained, local exhaust ventilation (LEV) is serviced and maintained when due and employees are trained in the use of available LEV to minimize exposure at work.

## 5. Conclusions

The IAQ of five workstations within NHPL was investigated, monitored, and measured. The major IAQ pollutants affecting the workstations are VOCs, temperature, and relative humidity. Meanwhile, for particulate matter, they are Na_2_O, Al_2_O_3_, SiO_2_, SO_3_, CaO and Fe_2_O_3_. This is so because the IAQ pollutants affecting the workstations have exceeded one or two PELs (especially VOCs and temperature) and are below the expected PELs, as in the case of the %RH of all the workstations. From this study, it is evident from Figure 3 that natural ventilation in buildings is important to supply fresh air to occupants, and to dilute and exhaust pollutants to provide acceptable IAQ. From the structures and activities of the workstations, source control/removal would be the most effective way to reduce indoor air (IA) pollutants, as agreed by another research study [27].

Based on the outcome of the study and related health effects associated with identified IA pollutants in the work environment, it was concluded that workplace health and safety measures that include the enforcement of personal protective equipment use, especially during handling of sensitizing compounds; health screening and periodic surveillance programs; as well as substitution of hazardous pollutants with safer compounds where possible should be encouraged as part of workplace safety and health management systems within the organization.

## Figures and Tables

**Table 1:** Information of the Monitored Workstations NHPL.

No.	Workstation	Floor	Floor Area (m^2^)	Minimum No. of Workforce per Shift	Maximum No of Workers per Shift
1	BPA	Ground floor	50	9	32
2	BOA	Ground floor	50	3	8
3	FFA	First floor	200	7	17
4	SPA	Ground floor	400	34	71
5	BSP	Ground floor	200	6	8

**Table 2:** Reported indoor air syndrome among employees of NHPL.

Workstation	Reported Exposure Health Complaints		
	Respiratory Related	Eyes Related	Other Surface Injuries
BPA (32)	7	10	0
BOA (8)	2	2	0
FFA (17)	4	3	1
SPA (71)	17	9	6
BSP (8)	2	3	0
Total	32	26	7

**Table 3:** Measured Indoor Air Quality at monitored Workstations within NHPL.

			Sampling Points			
Parameters	(BPA)	(BOA)	(FFA)	(SPA)	(BSP)	*p* Value
PM_2.5_ (µg/m^3^)	44 ± 5.66	24.5 ± 2.38	48.5 ± 6.25	28.75 ± 1.89	38.25 ± 4.11	S
PM_10_ (µg/m^3^)	54 ± 8.17	26.75 ± 2.75	61.75 ± 9.53	33.75 ± 3.95	45.25 ± 6.23	S
HCHO (mg/m^3^)	0.01 ± 0.02	0.0025 ± 0.00	0.0125 ± 0.00	0.00 ± 0.00	0.0025 ± 0.00	NS
VOCs (mg/m^3^)	1.35 ± 0.28	0.98 ± 0.40	0.93 ± 0.06	0.27 ± 0.04	0.09 ± 0.00	S
CO_2_ (mg/m^3^)	1227.75 ± 55.22	1265 ± 55.82	1190.25 ± 4.5	1155.25 ± 2.36	1137 ± 2.00	S
T (°C)	25.65 ± 0.42	27.6 ± 0.34	28.15 ± 0.10	26.95 ± 0.12	27.55 ± 0.12	S
RH (%)	20.13 ± 0.22	22.55 ± 0.20	22.7 ± 0.62	22.75 ± 0.17	23.80 ± 0.52	S

Note: The decision (*p* value), *p* > 0.05 is Not Significant (NS), *p* < 0.05 is Significant (S), *p* value at 95% confidence level.

**Table 4:** The AQ Parameters and Permissible Exposure Limits (8 h TWA).

	Sample Values		Regulatory Limits	
Parameters	Minimum	Maximum	OSHA	NESREA
PM2.5(µg/m^3^)	24.50	48.50	50.00	-
PM10(µg/m^3^)	26.75	61.75	150.00	180.00
HCHO (mg/m^3^)	0.00	0.01	0.75	0.10
VOCs (mg/m^3^)	0.09	1.35	0.75	0.60
CO_2_ (mg/m^3^)	1137	1265.00	5000.00	10,000.00
T (°C)	25.65	28.15	24.44	25.50
RH (%)	20.13	23.80	60.00	70.00

**Table 5:** Characterization of Particulate Matter.

Parameter (wt. %)	Sampling Location				
	BPA	BOA	FFA	SPA	BSP
Na_2_O	26.580	29.096	29.945	27.955	25.490
MgO	1.101	1.356	0.307	0.378	6.348
Al_2_O_3_	3.183	4.178	2.407	2.904	16.498
SiO_2_	41.722	40.711	26.193	25.279	25.788
P_2_O_5_	0.446	0.000	0.133	0.139	0.014
SO_3_	18.014	14.213	38.086	31.767	9.600
Cl^−^	0.544	0.567	0.674	0.341	0.834
K_2_O	0.075	0.017	0.111	0.113	0.150
CaO	6.406	9.287	1.657	2.025	14.311
TiO_2_	0.250	0.272	0.091	0.145	0.528
Cr_2_O_3_	0.008	0.002	0.002	0.015	0.003
Mn_2_O_3_	0.017	0.006	0.006	0.071	0.010
Fe_2_O_3_	1.629	0.279	0.376	8.834	0.402
ZnO	0.023	0.000	0.009	0.031	0.005
SrO	0.001	0.017	0.003	0.002	0.019

**Table 6:** Particulate Matter Characterization 8 h TWA.

Parameter (mg/m^3^)	Sampling Location					
	BPA	BOA	FFA	SPA	BSP	OSHA PEL
Na_2_O	22.46	24.59	25.30	23.62	21.54	15
MgO	0.61	0.75	0.17	0.21	3.49	15
Al_2_O_3_	4.42	5.81	3.34	4.04	22.93	15
SiO_2_	34.17	33.35	21.45	20.70	21.12	21.06
P_2_O_5_	0.86	0.00	0.26	0.27	0.03	15(NE)
SO_3_	19.67	15.52	41.57	34.68	10.48	15 (NE)
Cl^−^	0.26	0.27	0.33	0.17	0.40	15
K_2_O	0.10	0.02	0.14	0.15	0.19	15 (NE)
CaO	4.90	7.10	1.27	1.55	10.94	5
TiO_2_	0.27	0.30	0.10	0.16	0.57	15
Cr_2_O_3_	0.02	0.01	0.01	0.03	0.01	0.5
Mn_2_O_3_	0.00	0.00	0.00	0.02	0.00	5 (as Mn)
Fe_2_O_3_	3.55	0.61	0.82	19.23	0.88	10 (as fumes)
ZnO	0.03	0.00	0.01	0.03	0.01	15
SrO	0.00	0.03	0.00	0..00	0.03	15

## Data Availability

Data is contained within the article.
